# Supplementary material for: One Health approach for Brucella canis: Serological and molecular detection in animal-hoarding individuals and their dogs
Source: PLoS Negl Trop Dis. 2024 Mar 12;18(3):e0011974. doi: 10.1371/journal.pntd.0011974 (PMC10959369; doi:10.1371/journal.pntd.0011974)
Supplement: S1 Questionnaire — (DOCX) [file pntd.0011974.s001.docx]

**S1 Questionnaire: Epidemiological questionnaire used to investigate serological and molecular *Brucella canis* and associated factors in individuals with animal hoarding disorder and their dogs in Curitiba.**

**(Translated to English)**

Researcher: ____________________________________ Date: ___/___/_____ Case identification: _______________ Dog identification: _________________ Address: ________________________________________________________

**1.** Dog’s name:____________________________ **2.** Date**:** ____/____ /______

**3.** Coat type: _____________ **4.** Sex: ( )Female ( )Male ( )NA

**5.** Age:___B__ ( )NA

**6.** The animal is vaccinated: ( )No ( )Yes ( )NA

**7.** If yes, which vaccine?: ( )Polyvalent ( )Anti-rabies ( )NA

**8.** Body score: ( )Cachectic ( )Skinny ( )Normal ( )Fat ( )NA

**9.** Mucous membranes: ( )Normal ( )Hypocolored ( )Hypercolored ( )Congested

( )Icteric ( )NA

**10.** Ectoparasites: ( )No ( )Yes ( )NA

**10.1** If yes, which ones: ( )Flea ( )Tick ( )Larva

( )Others:______________________________________________ ( )NA

**11.** Skin lesions: ( )No ( )Yes ( )NA

**11.1** If yes, what is the characteristic of the lesions: ( )Dry ( )Ulcerative ( )Purulent

( )Others:___________________________________________

**11.2** Lesions suggestive of: ( )Scabies ( )Flea allergy dermatitis ( )Fight ( )Accident

( )Others:_________________

**12.** Nasal or eye discharge: ( )No ( )Yes ( )NA,

**12.1** If yes, what is the characteristic: ( )Mucous ( )Purulent

( )Others:_____________________________ ( )NA

**13.** Itching: ( )No ( )Yes ( )NA

**14.** Anal and perianal region dirty with diarrhea residue: ( )No ( )Yes ( )NA

**15.** When the interviewer approaches, the animal: ( )Run away ( )Growls ( ) Put the tail between the legs ( )Approaches ( )Jump ( )No reaction

( )Others:________________ ( )NA

**16.** When the owner approaches, the animal: ( )Run away ( )Growls ( ) Put the tail between the legs ( )Approaches ( )Jump ( )No reaction

( )Others:________________ ( )NA

**17.** Living place: ( )Freely in the yard ( )Inside home ( )Leashed ( )Individually kenneled( ) Collectively kenneled ( )Cagelly ( )Others:________ ( )NA
