# Supplementary material for: One Health approach for Brucella canis: Serological and molecular detection in animal-hoarding individuals and their dogs
Source: PLoS Negl Trop Dis. 2024 Mar 12;18(3):e0011974. doi: 10.1371/journal.pntd.0011974 (PMC10959369; doi:10.1371/journal.pntd.0011974)
Supplement: Attachment — Submitted filename: Response letter_Brucella hoarders Dec22nd23.docx [file pntd.0011974.s002.docx]

Curitiba, December 22^nd^, 2023

**Response letter**

To: Joseph M. Vinetz
Section Editor

Re: Decision on your PLOS Neglected Tropical Diseases submission (PNTD-D-23-01267) - [EMID:fcb3018c5e7a2000]

Dear Editor,

Please find attached our reviewed manuscript entitled “One Health approach on *Brucella canis*: serological and molecular detection in animal hoarding individuals and their dogs”. All the corrections and suggestions indicated by the reviewers were fully addressed, accepted, and rewritten.

A response letter has been added to pinpoint the editor and reviewers’ requests, with a detailed list of corrections and a description of changes made within the manuscript. To better identify the corrections of each reviewer, changes were highlighted in different colors in the revised manuscript. Thank you for the opportunity and please do not hesitate to contact us with any further questions.

Sincerely,

Alexander Welker Biondo, DMV, MSc, PhD.

Professor, Department of Veterinary Medicine, UFPR, Brazil

Visiting Professor, Purdue University, IN, USA

E-mail: [abiondo@ufpr.br](about:blank) / Phone: +55 (41) 3350-5812

Reviewer 1 (yellow intext)

Reviewer #1: -Are the conclusions supported by the data presented? Partly

**Changed:** Conclusions were changed to clarify.

**Now you read: “**The present study is the first to assess brucellosis in a vulnerable population as a silent disease based on the environment and susceptible population. Despite the absence of seropositivity in individuals with AHD and the comparatively low seroprevalence in dogs, *B. canis* circulation and outbreaks should be considered in such human populations due to the high burden and recurrent character of *B. canis* exposure in high-density dog populations and the constant introduction of susceptible animals. In conclusion, serological and molecular assessments of vulnerable populations, particularly those with silent diseases, may serve as the basis for public health policies, including systematic diagnosis, control, monitoring, and prevention.” (Lines 318-327)

Reviewer #1: The study has serious limitations.

Firstly, the human samples are insufficient, while the dog samples are incompletely described. The authors should include a comprehensive table that includes number of dogs present in the household, number of dogs sampled, number of people living in the household and number of sampled people (including sex and age).

**Changed:** Reviewer is right. The human sample size was inserted as limitations. Table 2 was changed and comprehensive information about dogs was inserted in both table and in results section.

**Now you read:**

“As a limitation of this study, no sampling was performed for tissues, saliva, nasal or genital secretions, or urine, particularly following reproductive problems, which would have increased the diagnostic sensitivity [16]. Furthermore, access to and monitoring of households of individuals with AHD was limited by the classic “refusal to receive help” characteristic of the AHD population, which also impacted the low sampling of individuals with AHD in this study. Furthermore, the sampling dates used were different for dogs and individuals with AHD in compliance with different time permits from the ethics committee of animal use and the city secretary of health, which may have impacted the results due to potential dynamics over time in *B. canis* exposure. In addition, the epidemiological questionnaires failed to include important questions on associated risk factors for *B. canis* exposure, such as serology following reproductive failure or molecular testing of abortion tissues.” (Lines 303-315)

**“**The majority of the tested individuals with AHD were women (13/19), most of whom were elderly (9/13), with ages ranging from 66 to 86 years.” (Lines 218-219)

“Overall, seropositive dog samples were equally distributed between males and females (six each), with ages ranging from 1 to 16 years and dogs mostly living freely in the yard (9/12). In one household, two seropositive dogs lived in a collective kennel (Table 2).” (Lines 220-223).

**Table 2.** Distribution and frequency of presence of anti-*B. canis* antibodies in dogs and epidemiological data from dogs and individuals with AHD per household case. (Line 224)

Secondly, the authors mention questionnaires both in methods and discussion, but do not provide any data in the results section. Was there any statistical analysis performed on them? The questionnaire should be included as supplementary material

**Changed:** Due to low seroprevalence, only descriptive statistics were included. The epidemiological questionnaire was inserted as supplementary material as requested.

**Now you read:**

“Supplementary Questionnaire 1: Epidemiological questionnaire used to investigate serological and molecular *B. canis* and associated factors in individuals with animal hoarding disorder and their dogs in Curitiba.” (Lines 329-331)

Seropositivity does not in any way indicate an active disease.

**Changed:** Reviewer is right and the full text was properly corrected. Only exposure to *Brucella canis* was accessed herein. Thus, “canine brucellosis” was replaced by “*Brucella canis* exposure” throughout the manuscript.

**Now you read:**

“Short title: *Brucella canis* exposure in animal hoarders.” (Line 4)

“due to the high burden and recurrent character of *B. canis* exposure in high-density dog populations and the constant introduction of susceptible animals.” (Lines 48)

“**Table 1.** Detection of anti-*B. canis* antibodies by 2ME-MAT and LFIA and *Brucella* spp. DNA by PCR in human and dog samples.” (Lines 215-216)

“The present study is the first comprehensive investigation of *B. canis* exposure in.” (Lines 232)

“Serological diagnosis of *B. canis* exposure may be even more relevant in this study,” (Line 245)

“which may indicate that large unassisted dog populations living in confined places contributed to the transmission of *B. canis*, similarly observed in overpopulated kennels [17,30].” (Line 271)

“of *B. canis* circulation in high-density dog populations and the constant introduction of susceptible animals.” (Lines 275-277)

“In addition, the epidemiological questionnaires failed to include important questions on associated risk factors for *B. canis* exposure,” (Lines 314)

“should be considered in such human populations due to the high burden and recurrent character of *B. canis* exposure in” (Line 323)

Finally, a thorough revision of English language is required

**Changed:** The manuscript was revised by professional English editing service, and certified was following inserted.
